# The time-series behavior of neutrophil-to-lymphocyte ratio is useful as a predictive marker in non-small cell lung cancer

**DOI:** 10.1371/journal.pone.0193018

**Published:** 2018-02-15

**Authors:** Tatsunori Kiriu, Masatsugu Yamamoto, Tatsuya Nagano, Daisuke Hazama, Reina Sekiya, Masahiro Katsurada, Daisuke Tamura, Motoko Tachihara, Kazuyuki Kobayashi, Yoshihiro Nishimura

**Affiliations:** Division of Respiratory Medicine, Department of Internal Medicine, Kobe University Graduate School of Medicine, Kobe, Japan; University of North Carolina at Chapel Hill School of Medicine, UNITED STATES

## Abstract

**Background:**

Nivolumab improves the survival of advanced non-small cell lung cancer (NSCLC), but a significant number of patients still fail to benefit from this treatment. In this study, we evaluated the efficacy of the time-series behavior of neutrophil-to-lymphocyte ratio (NLR) in a complete blood count from advanced NSCLC patients as a predictive marker of the anticancer effect of nivolumab.

**Methods:**

We performed a retrospective review of medical records and collected data on patients with advanced NSCLC treated with nivolumab as second- and further-line treatments from December 2015 to March 2017. The NLRs were calculated before each treatment cycle for four cycles. These parameters were tested for its association with the overall survival (OS), progression-free survival (PFS) and time to treatment failure (TTF).

**Results:**

Nineteen patients were treated with nivolumab. Stratified by the response to nivolumab, the median OS was 2.8 months in progressive disease (PD) and 14.0 months in non-PD (p = 0.002). Before discontinuation of PD or toxicity, an NLR is rising from baseline in 5 out of 7 patients with PD and all of 4 patients with discontinuation due to toxicity. Patients with an >30% increase in NLR were associated with a significantly shorter TTF compared with those with stable or decrease in NLR both after first cycle (p = 0.014) and second cycle (p < 0.001).

**Conclusions:**

The NLR is suggested to be useful not only as a prognostic marker but also as a predictive marker for treatment with nivolumab. Further prospective study is warranted to develop a predictive algorithm to detect PD cases as early as possible by focusing the time-series behavior of NLR.

## Introduction

Cancer immunotherapy is a new strategy for advanced non-small cell lung cancer (NSCLC). Anti-programmed death-1 (PD-1) antibodies, such as nivolumab and pembrolizumab, inhibit PD-1-mediated signaling by blocking programmed death-ligand-1 (PD-L1) from binding to PD-1, thereby allowing T-cell activation and immune system recognition. These antibodies restore the patient’s natural tumor-specific T-cell-mediated immune responses.

In phase III trials, treatment with nivolumab for advanced NSCLC that had progressed during or after platinum-based chemotherapy improved the overall survival (OS) compared to treatment with docetaxel [1,2]. In addition, some patients show a durable clinical benefit by nivolumab treatment. Nivolumab is now commonly used for advanced NSCLC; however, the mechanism underlying its effect are not fully known to develop predictive markers.

The progression-free survival (PFS) curves in treatment with nivolumab are overlapping for several months after starting treatment and showing that considerable numbers of patients do not respond to the treatment from the beginning [1,2], therefore it is necessary to quickly find non-responders in these treatments. Our clinical question is how to distinguish between responders and non-responders as early as possible during treatment with nivolumab.

Recently, the tumor microenvironment, which is largely maintained by inflammatory cells, is thought to be an indispensable participant in the neoplastic process, fostering proliferation, the tumor survival and migration [3]. The role of inflammation has been shown to be important in tumorigenesis, and an inflammatory microenvironment was found to be a necessary component in NSCLC [4]. Blood-based inflammatory parameters, such as the neutrophil-to-lymphocyte ratio (NLR) and the platelet-to-lymphocyte ratio (PLR), have been reported to predict the prognosis in solid tumors. NLR has been suggested as a simple index of the systemic inflammatory response in critically ill patients [5]. A previous study found that a baseline NLR ≤ 5, which is commonly used as a threshold of the NLR, was associated with an improved survival in patients treated with nivolumab [6]. In addition, a baseline PLR ≤ 262 has also been shown to improve survival as well [7]. Although the baseline NLR and PLR are useful for stratifying patients before nivolumab, predictive biomarkers for determining whether nivolumab should be continued is still unknown.

In contrast, a high posttreatment NLR was reported to be associated with a poor prognosis in never-smokers with advanced lung adenocarcinoma that had been treated with gefitinib and gemcitabine plus cisplatin as first-line therapy [8]. Given that nivolumab modifies the immunological status, we hypothesized that the time-series behavior of the NLR reflects and predicts tumor responses. There have been no reports focusing on the time-series behavior of the NLR during treatment. Our study reports for the first time a connection between the time-series behavior of the NLR and clinical benefit in advanced NSCLC treated with nivolumab.

In the present study, we aimed to determine whether the time-series behavior of NLR are predictive markers of the anticancer effect of nivolumab for patients with previously treated NSCLC.

## Materials and methods

### Patients and treatments

We performed a retrospective review of electronic medical records and collected data on patients who had been diagnosed with NSCLC histologically or cytologically and treated with nivolumab as monotherapy from December 2015 to March 2017 at Kobe University Hospital.

The data collected from all of the patient medical records included the following: gender, age, smoking history, Eastern Cooperative Oncology Group Performance Status (ECOG PS) at time of initiating the treatment, histology, clinical or pathological stage based on the seventh edition of *TNM classification of Malignant Tumours* by the International Union Against Cancer and the American Joint Committee on Cancer, molecular profiling for epidermal growth factor receptor (EGFR), anaplastic lymphoma kinase (ALK), and proto-oncogene tyrosine kinase c-ROS1 (ROS1), lines of prior therapy and follow-up status. Nivolumab was administered intravenously at doses of 3 mg/kg as one cycle every 2 weeks.

This retrospective analysis was approved by the Institutional Review Board of Kobe University Hospital (#170061), and all patients signed their comprehensive written informed consent before undergoing treatment. All data were fully anonymized before we assessed.

### Neutrophil-to-lymphocyte ratio (NLR)

We recorded time-series laboratory data before the initiation of each treatment cycle with nivolumab for four cycles. The NLR was defined as absolute neutrophil count (ANC) divided by absolute lymphocyte count (ALC).

The baseline NLR (NLRpre) was determined using the complete blood count (CBC) measured before the first cycle of treatment. The posttreatment NLR (NLRpost) was determined using the CBC measured on day 1 of the second cycle of treatment. The time-series NLR (NLRseries) during the treatment was determined using the CBC measured on day 1 of each next cycle. To evaluate the NLRseries, we checked the NLR 3–5 weeks before treatment (NLRformer). We considered the influence of chemotherapy-induced toxicities, therefore we avoided the period during bone marrow suppression to obtain NLRformer. We divided NLRseries into two groups of > 30% increase in NLR group (iNLR) and stable or decrease in NLR group (sNLR) at the time after first or second cycle by the percentage change from NLRpre value.

### Statistical analyses

OS was defined as the time from date of treatment initiation to the date of death due to any cause. Patients who were still alive were censored at the last follow-up. PFS was defined as the time from the date of treatment initiation until progression, as documented by imaging, according to Response Evaluation Criteria In Solid Tumors (RECIST) Version 1.1 or clinical examination or death. Time to treatment failure (TTF) was defined as the time from date of treatment initiation to the date of disease progression, death, treatment discontinuation, or initiation of a new antitumor therapy. Those who were still alive and free from progression were censored at the last follow-up. Response to therapy was assessed by the treating physician and was classified as complete response (CR), partial response (PR), stable disease (SD), progressive disease (PD) or not evaluable (NE).

All statistical analyses were performed using EZR (Saitama Medical Center, Jichi Medical University, Saitama, Japan, version 1.35), which is a graphical user interface for R (The R Foundation for Statistical Computing, Vienna, Austria, version 3.3.2) [9]. The OS, PFS and TTF curves were estimated using the Kaplan-Meier method and compared using the log-rank test. *P <* 0.05 was considered statistically significant in all analyses.

## Results

### Patient characteristics

We performed a retrospective review of 20 patients who had been diagnosed with NSCLC histologically or cytologically and treated with nivolumab as monotherapy from December 2015 to March 2017 at Kobe University Hospital. We excluded one patient who treated as first-line chemotherapy for recurrence after adjuvant chemotherapy. Nineteen patients had been treated with nivolumab. The baseline characteristics of the patients included in the final analysis are shown in Table 1. The median of the treatment cycle was 3 (range, 1–24; interquartile range [IQR], 2–6). The overall response rate was 26.3% (5 of 19 patients).

**Table 1:** Patient characteristics and clinicopathological data.

Patients		(N = 19)
Gender		
	Male	19
	Female	0
Age, years		
	Median (Range)	70.5 (41–83)
ECOG PS		
	0	0
	1	19
	2	0
Histology		
	Adenocarcinoma	10
	Squamous cell carcinoma	6
	Others	3
Tumor Stage		
	Stage III	5
	Stage IV	7
	Recurrent	7
Lines of prior therapy		
	1	5
	2	9
	3	4
	4	1
Targetable driver mutations		
	EGFR	1
	ALK	0
	ROS1	0
Smoking history		
	Heavy smoker	18
	Light smoker	1
	Never smoker	0
Neutrophil-lymphocyte ratio (NLRpre)		
	> 5	6
	≤ 5	13
Response		
	PR	5
	SD	7
	PD	7

EGFR, epidermal growth factor receptor; ALK, anaplastic lymphoma kinase; ROS1, proto-oncogene tyrosine kinase c-ROS1; PR, partial response; SD, stable disease; PD, progressive disease.

### Overall survival

**Fig 1:**
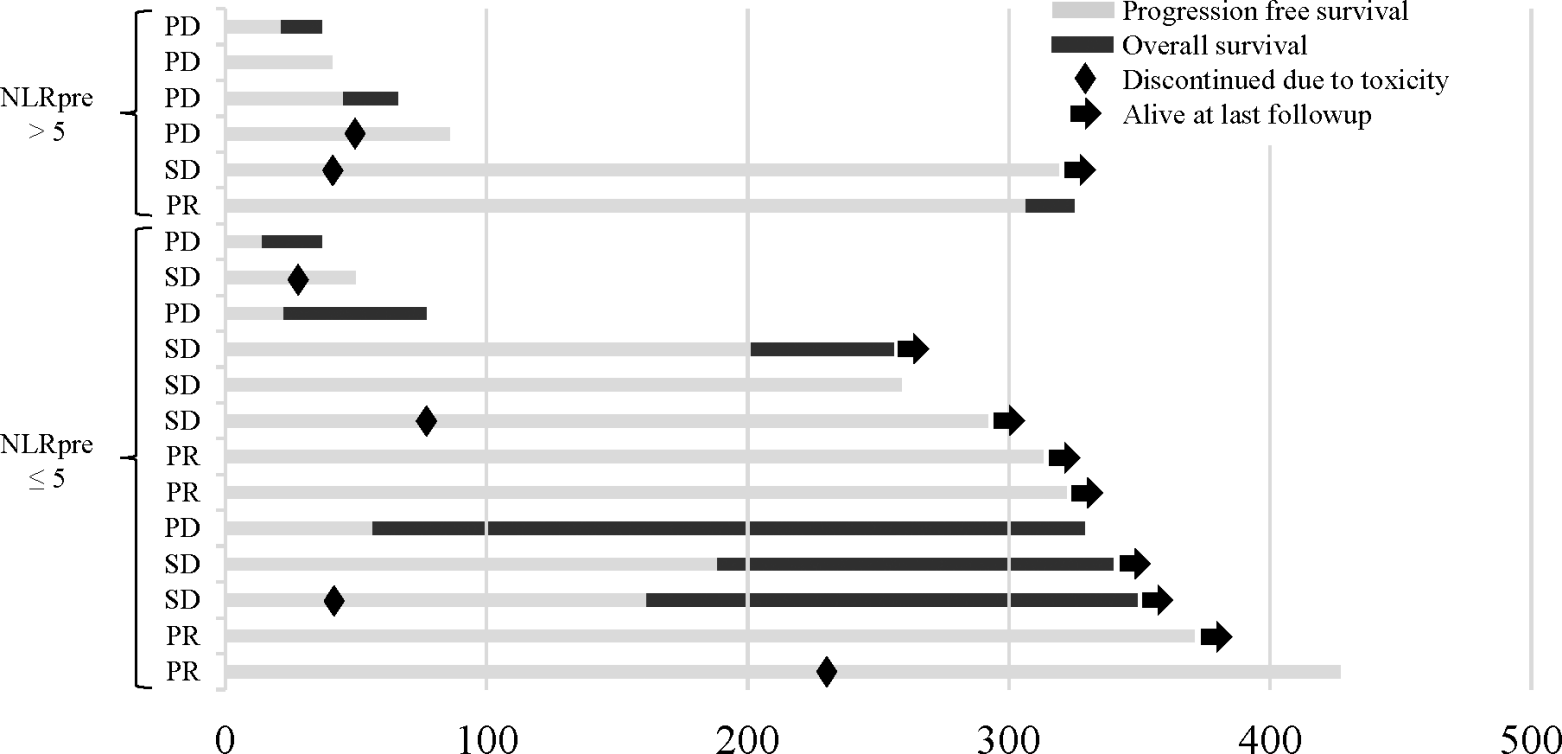
Swimmers plot detailing progression survival (PFS) and overall survival (OS). Swimmers plot illustrating overall survival of all individual patients in this study. Survival is divided into two periods: PFS (gray) and OS (black). →, alive at last follow-up; ◆, discontinued due to toxicity.

**Fig 2:**
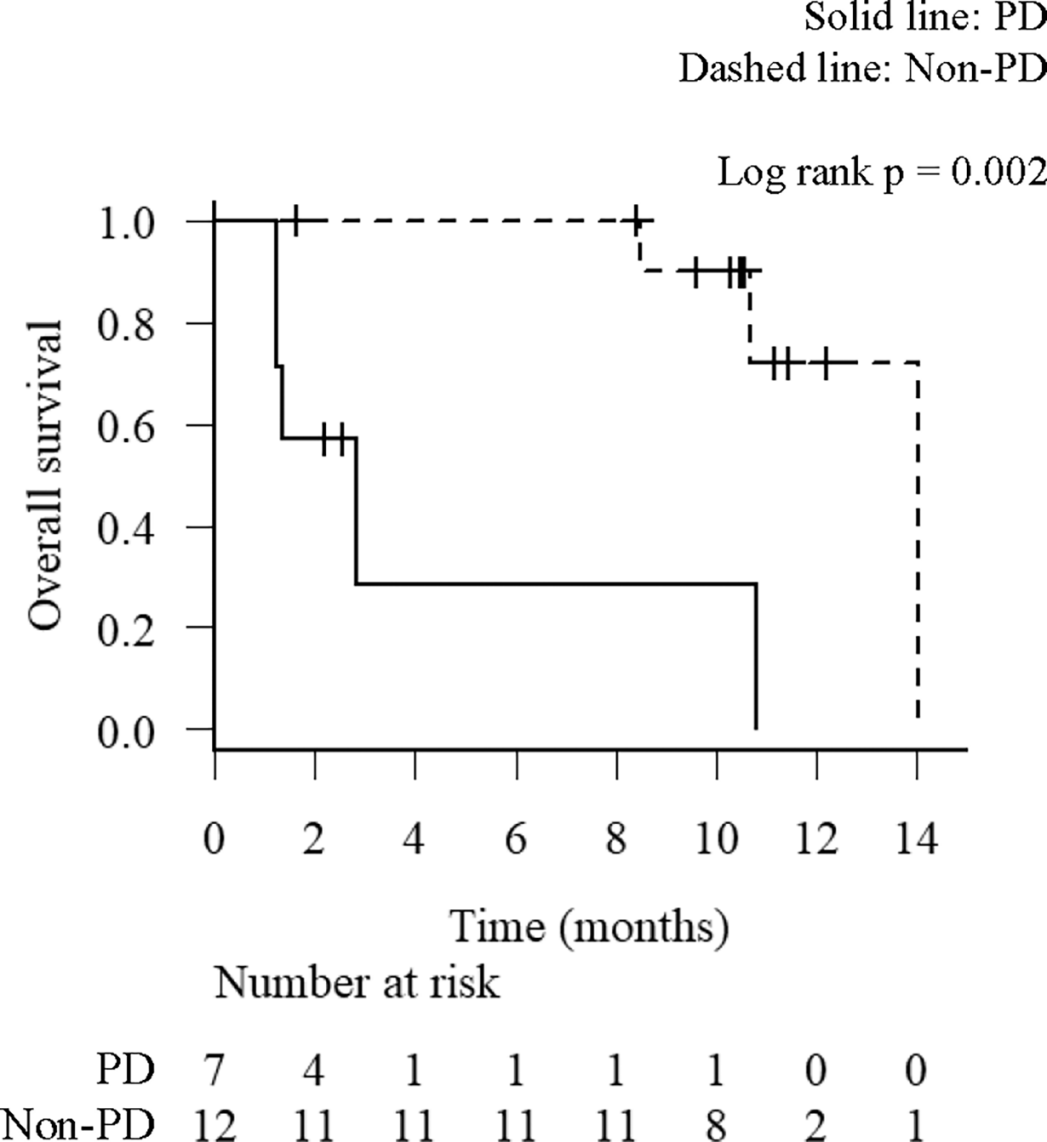
Overall survival (OS) analysis by the response to nivolumab. Kaplan-Meier survival curves for the OS according to the response to nivolumab. Solid line, progressive disease (PD); Dashed line, stable disease (SD) and partial response (PR).

The median OS was 10.8 months in the total population. Swimmers plot illustrating OS of all individual patients in this study is shown in Fig 1. Durable responses are observed in patients with responses of PR or SD including patients who discontinued nivolumab due to severe toxicities. Stratified by response to nivolumab, the median OS was 2.8 months in PD and 14.0 months in non-PD (p = 0.002) (Fig 2). These results indicate that patients with tumor response to nivolumab have a significantly longer OS.

### Baseline NLR (NLRpre)

The median values for baseline ANCs and neutrophil proportions were 4914 /μL (range, 3120–11310) and 67.2% (range, 57.6–88.0), respectively. The median values for baseline ALCs and lymphocyte proportions were 1532 /μL (range, 216–2628) and 19.0% (range, 2.8–29.9), respectively.

**Fig 3:**
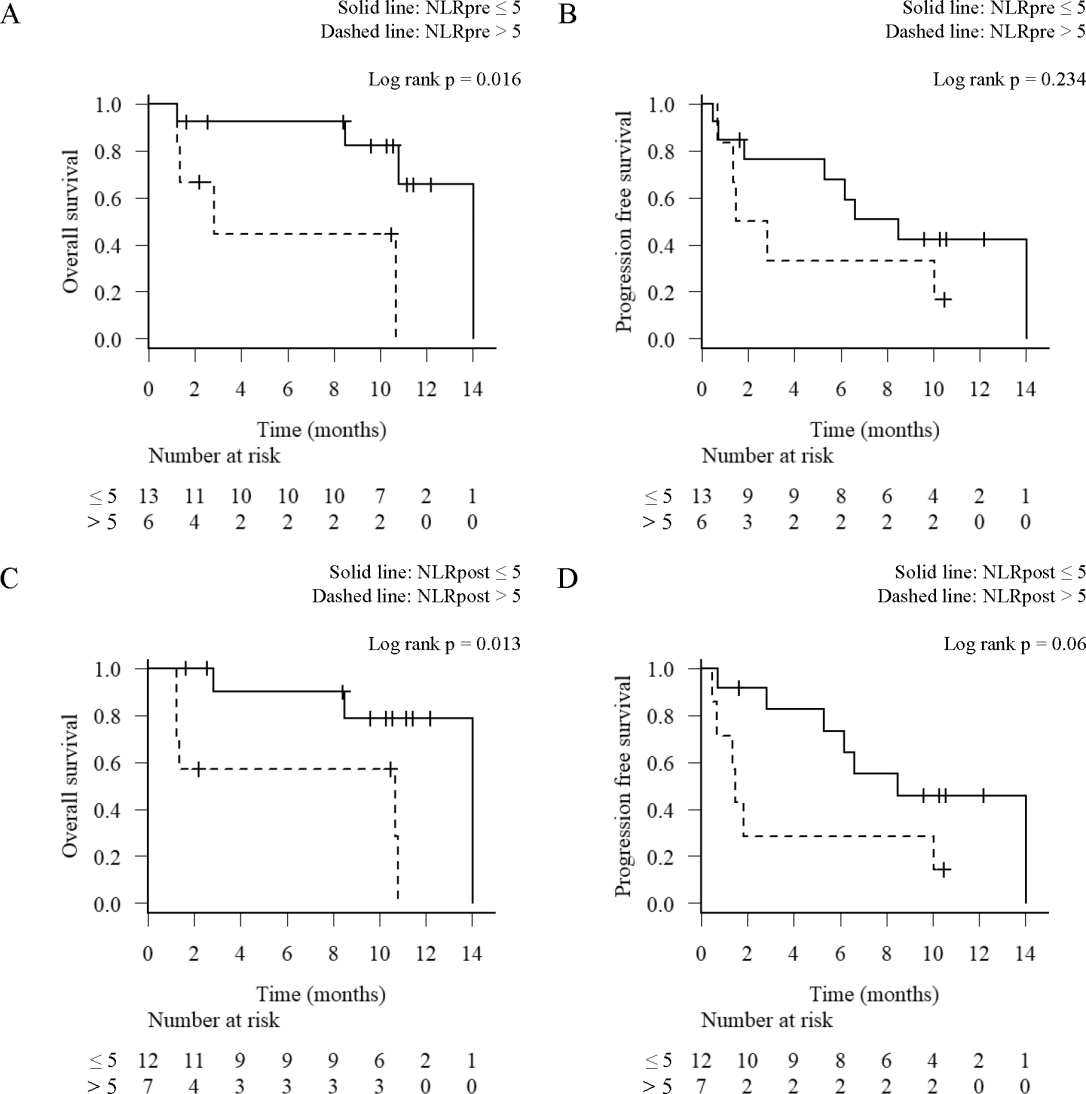
Overall survival (OS) and progression-free survival (PFS) analysis by the baseline (NLRpre) and post-treatment neutrophil-to-lymphocyte ratio (NLRpost). Kaplan-Meier survival curves for the OS (A, C) and PFS (B, D) according to the level of NLRpre (A, B) and the NLRpost (C, D). Solid line, NLR ≤ 5; Dashed line, NLR > 5.

The median NLRpre was 3.5 (range, 1.9–31.4; IQR, 2.8–5.6). The NLRpre was > 5 in 6 patients (31.6%) and ≤ 5 in 13 patients (68.4%). The median OS for patients with NLRpre > 5 and ≤ 5 was 2.8 and 14.0 months (p = 0.016) (Fig 3A), respectively. The median PFS for patients with NLRpre > 5 and ≤ 5 was 2.2 and 8.5 months (p = 0.234) (Fig 3B), respectively. The NLRpre > 5 was significantly associated with a shorter OS compared with the NLRpre ≤ 5 (p = 0.016). However, tumor responses were observed in some of the patients in NLRpre > 5 and non-responses were observed in NLRpre ≤ 5 (Fig 1). These results suggest that while the NLRpre was useful as a prognostic marker as reported previously, there was a limitation to distinguish between responders and non-responders to nivolumab.

### Post-treatment NLR (NLRpost)

The median NLRpost was 4.3 (range, 1.5–20.8; IQR, 3.1–7.1). The NLRpost > 5 was observed in 7 patients (36.8%), whereas the NLRpost ≤ 5 was observed in 12 patients (63.2%). The median OS for patients with the NLRpost > 5 and ≤ 5 was 10.7 and 14.0 months (p = 0.013) (Fig 3C), respectively. The median PFS for patients with the NLRpost > 5 and ≤ 5 was 1.5 and 8.5 months (p = 0.06) (Fig 3D), respectively. These results showed that NLRpost > 5 was associated with shorter OS.

### Time-series behavior of NLR (NLRseries)

**Fig 4:**
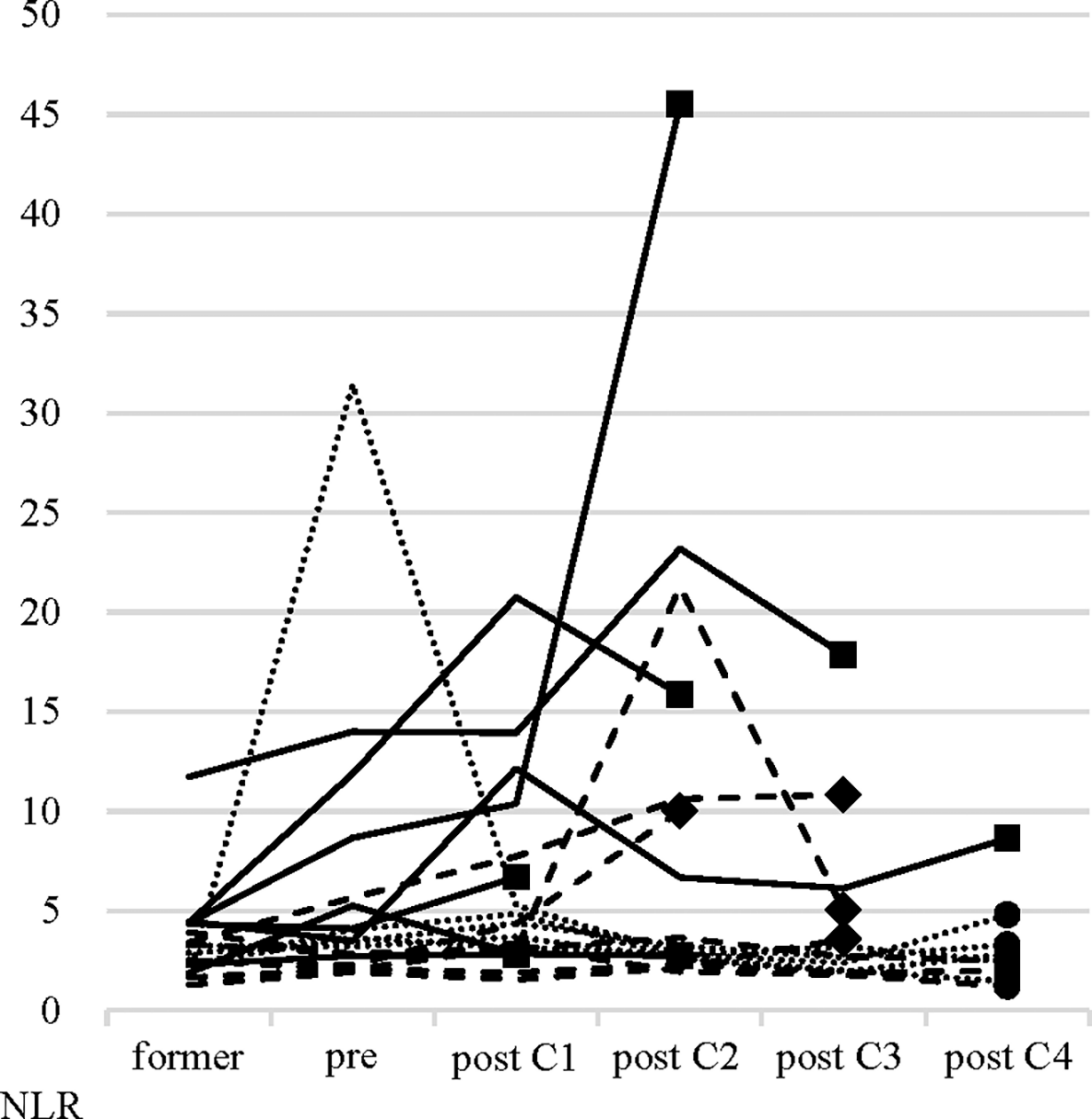
The time-series behavior of neutrophil-to-lymphocyte ratio (NLRseries). NLRseries in patients receiving nivolumab. Solid line, progressive disease (PD); Dashed line, stable disease (SD); Dotted line, partial response (PR); ●, continued treatment; ◆, discontinued due to toxicity; ■, progressive disease.

The NLRseries in nineteen patients is shown in Fig 4. There was only 1 patient (5.3%) with NLRformer > 5. On the other hand, the number of patients with NLRpre > 5 increased up to 6 (31.6%). These results support that an NLR changed over time and that NLRseries reflected disease conditions. Before discontinuation of PD or toxicity, an NLR is rising from NLRpre in 5 out of 7 patients with PD and all of 4 patients with discontinuation due to toxicity. Among cases where treatment was terminated due to PD or toxicity at an early stage, there were cases in which NLR is remarkably increased followed by decreased before discontinuation of treatment.

**Fig 5:**
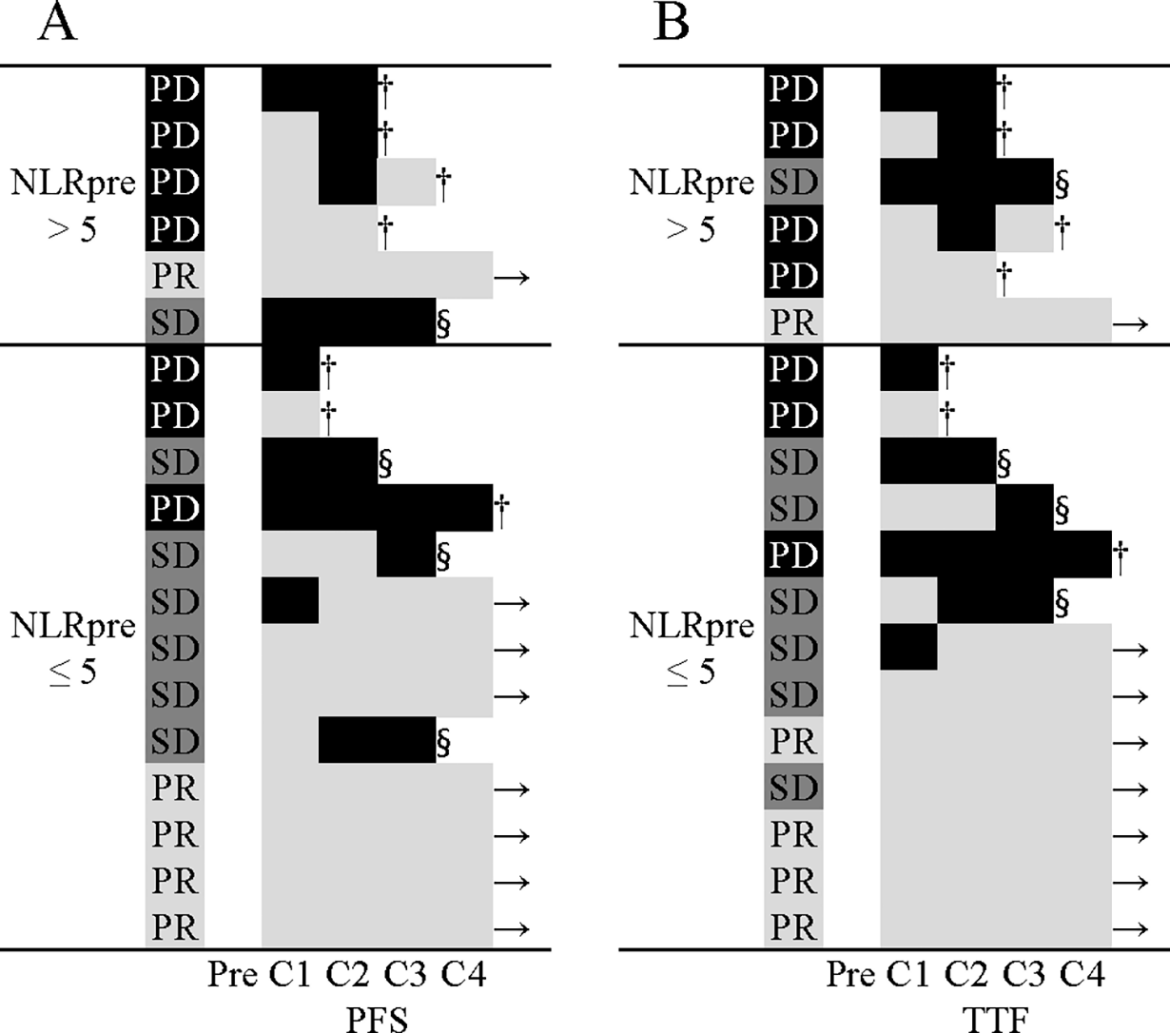
Swimmers plot detailing progression-free survival (PFS) and time to treatment failure (TTF) by the time-series behavior of neutrophil-to-lymphocyte ratio (NLRseries). Swimmers plots for PFS (A) and TTF (B) time from treatment started to the fourth cycle of treatment. NLRseries is divided into two groups of > 30% increase in NLR group (black) and stable or decrease in NLR group (gray). →, continued treatment; §, discontinued due to toxicity; †, progressive disease; Pre, pretreatment; C1, first cycle; C2, second cycle; C3, third cycle; C4, fourth cycle.

The PFS and TTF were arranged in ascending order in the NLRpre > 5 and ≤ 5, respectively (Fig 5). There is 1 patient of NLRpre ≤ 5 with iNLR followed by sNLR. In this patient, NLR increased from 2.0 to 3.2, then decreased, but no infection, PD, pseudo-progression or toxicity were noted. Patients with iNLR tended to have a shorter PFS and TTF compared with those with sNLR.

**Fig 6:**
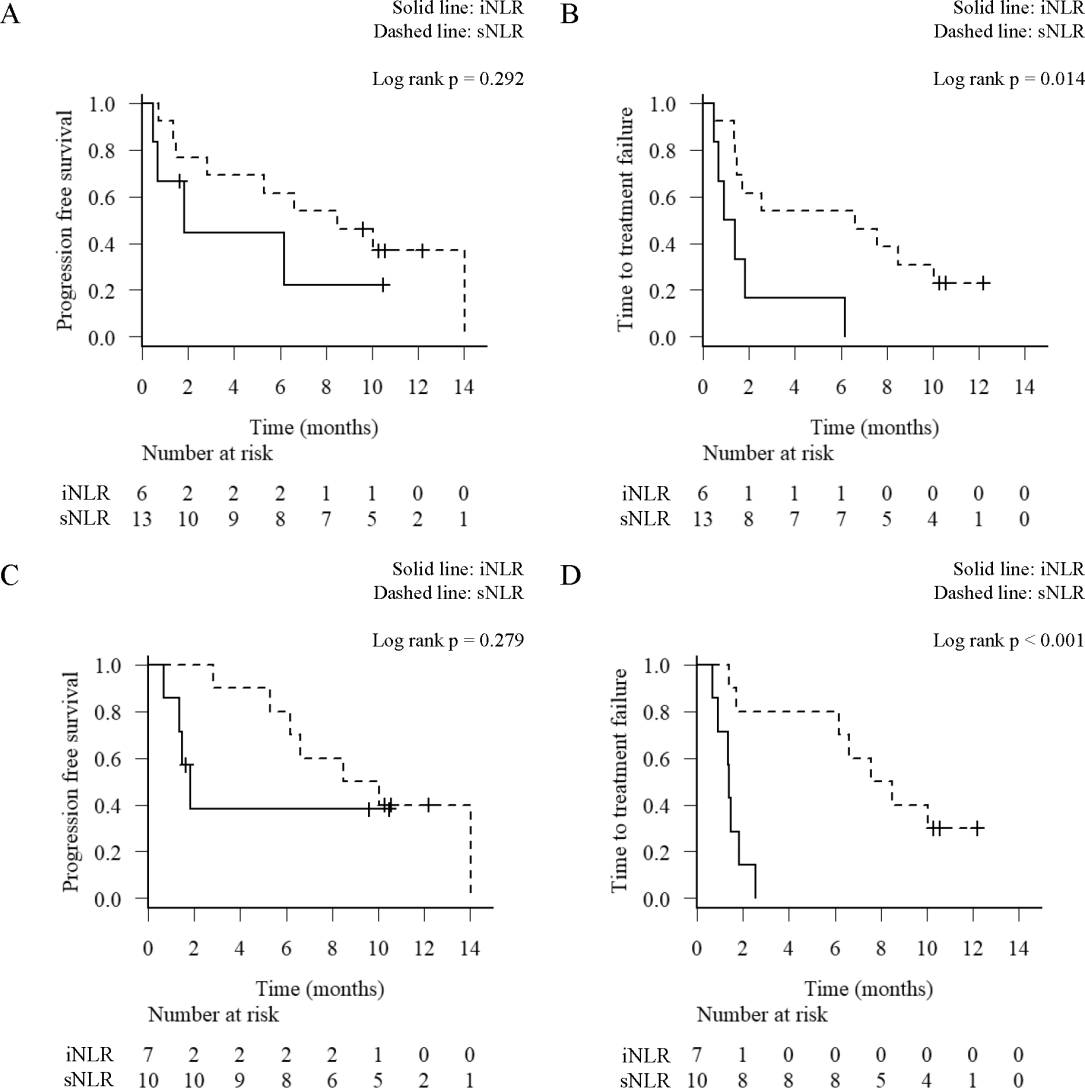
Progression-free survival (PFS) and time to treatment failure (TTF) analysis by the time-series behavior of neutrophil-to-lymphocyte ratio (NLRseries). Kaplan-Meier survival curves for the PFS (A, C) and TTF (B, D) according to the level of NLRseries (A, B) after the first cycle of treatment and (C, D) after the second cycle of treatment. Solid line, an increase of >30% NLR (iNLR); Dashed line, stable or decrease NLR (sNLR).

There were 6 (31.6%) in iNLR and 13 (68.4%) in sNLR after first cycle of treatment. The median PFS for patients with iNLR and sNLR was 1.8 and 8.5 months (p = 0.292) (Fig 6A), respectively. The median TTF for patients with iNLR and sNLR was 1.1 and 6.6 months (p = 0.014) (Fig 6B), respectively.

There were 7 (41.2%) in iNLR and 10 (58.8%) in sNLR after second cycle of treatment. The median PFS for patients with iNLR and sNLR was 1.8 and 9.3 months (p = 0.279) (Fig 6C), respectively. The median TTF for patients with iNLR and sNLR was 1.4 and 8.5 months (p < 0.001) (Fig 6D), respectively.

The TTF of iNLR was a significantly shorter compared with that of sNLR both after first cycle (p = 0.014) and second cycle (p < 0.001). On the other hand, there was no significant difference in PFS after either first or second cycle between the patients with iNLR and sNLR. As shown in Fig 5, iNLR closely related to therapy discontinuation due to PD and toxicity.

## Discussion

Our results indicate that the NLR is useful not only as a prognostic marker but also as a predictive marker for treatment with nivolumab. To our best knowledge, this is the first report to associate the NLRseries with clinical outcomes including TTF of treatment with nivolumab.

In the previous study, a high NLRpre was reported to be associated with a poor outcome for various types of cancers [10]. Some thresholds of NLR for patients with NSCLC have been proposed (Table 2) [6–8,11–17]. Further studies will be needed to determine the actual variety of these thresholds.

**Table 2:** Studies of the efficacy of the baseline NLR for advanced NSCLC.

Treatment	Number (Male/Female)	Histology (Ad/Sq/Others)	Cut off	Analysis	References
CT	388 (276/112)	274/76/38	Quartile	OS/PFS	[11]
CT	171 (143/28)	69/31/71	5	OS/PFS	[12]
CT or TT	199 (17/182)	199/0/0	3.25	OS/PFS	[8]
CT(+BEV)	73 (55/18)	58/11/4	4	PFS	[13]
CT	156 (80/76)	44/85/21	5	OS	[14]
CT	182 (119/63)	128/48/6	2.63	OS/PFS	[15]
TT	81 (47/32)	48/33/0	3.5	OS/PFS	[16]
CT or TT	401 (275/126)	258/ 94/ 49	3.7	OS/PFS	[17]
IO	175 (95/80)	133/42/0	5	OS/PFS	[6]
IO	52 (29/23)	30/18/4	5	OS	[7]

CT, chemotherapy; TT, targeted therapy; BEV, bevacizumab; IO, immune-oncology; OS, overall survival; PFS, progression-free survival.

In the present study, we showed that an iNLR reflect therapy failure, whereas sNLR reflect therapy success. PD cases are shorter OS than non-PD cases, it is important to find PD cases as early as possible. Our results are consistent with the findings in the clinical studies which were done in the development of nivolumab [1,2] and show that durable responses are observed in some patients. These findings give a clue to distinguish PD cases as early as possible. Therefore, the early appearance of iNLR may be helpful to find disease progression or serious adverse events and not to miss the timing to switch the next treatment. Together, these results suggest that the NLRseries may be useful as predictive markers in immunotherapy.

It has been reported that interleukin (IL)-17 producing T cells release C-X-C motif chemokines that migrate neutrophils, which may induce increase NLR, as well as promote differentiation into peritumor macrophages (TAMs) [18]. Elevated NLR was correlated with tumor progression and antitumor T cell exhaustion via an inflammatory tumor microenvironment provided by IL-17 producing T cells and TAMs [18]. Inhibition of IL-17A changed the balance of T cells within the tumor and the expression of other cytokines [19]. A recent study in mouse model suggested that an increase in IL-17A promote tumor progression by promoting inflammation mediated by cytokines and chemokines (IL-6, granulocyte colony-stimulating factor [G-CSF], C-X-C motif chemokine ligand 1 and milk fat globule-EGF factor 8 protein) and that IL-17A may be involved as one of the mechanisms of resistance to PD-1 antibody [20]. IL-17 is a potent activator of neutrophils through regulation of G-CSF and the G-CSF receptor [21]. The NLRseries may reflect the balance of T cells including T-helper-17 and the expression of cytokines mediated by IL-17, indirectly indicating resistance to nivolumab.

Results from a phase III trial of pembrolizumab as first-line therapy for advanced NSCLC showed an improved OS and PFS of patients compared with platinum-based chemotherapy [22]. In phase III trial, treatment with pembrolizumab for advanced NSCLC that had progressed after at least platinum-based chemotherapy improved the OS compared to treatment with docetaxel [23]. These results have been shown that the expression of PD-L1 can be a powerful biomarker. The PD-L1 immunohistochemistry assay with the murine 22C3 anti-human PD-L1 antibody has been approved for selecting patients likely to be responsive to pembrolizumab in Japan. However, some patients do not respond to pembrolizumab even if their PD-L1 expression is positive. Therefore, identifying a novel predictive marker for immune therapy benefits is a vital task for clinicians.

Biomarkers for cancer immunotherapy are currently being explored. CD8-positive tumor-infiltrating lymphocytes (TILs) have been reported as a predictive marker for treatment with pembrolizumab in patients with advanced melanoma [24]. PD-L1 on tumors interacts with PD-1 on the surface of CD8-positive TILs, so the PD-1 inhibitory pathway regulates the tumor-infiltrating CD8 T-cell responses [25]. The nonsynonymous mutation burden was reported to be associated with a durable clinical benefit of treatment with pembrolizumab in patients with NSCLC [26]. However, these reported biomarkers are limited to precisely distinguish between responders and non-responders.

The fact that our study was single-institutional, retrospective, small-sized and biased patient characteristics may make it difficult to generalize the result. We did not measure the PD-L1 status for the patients included in this study, therefore we could not consider the correlation with the PD-L1 status. Early in treatment with nivolumab, it may be possible to distinguish between responders and non-responders by paying attention to the patient’s immune status a few days after treatment initiation. Future studies will validate the expression of cytokines and the efficacy of the time-series behavior of NLR.

In conclusion, the NLR is suggested to be useful not only as a prognostic marker but also as a predictive marker for treatment with nivolumab. The NLRseries might be an effective marker to detect therapy discontinuation due to toxicity and progressive disease as early as possible.
